# Política Nacional de Atenção às Cardiopatias Congênitas: Reflexões e Propostas para o Fortalecimento da Assistência no Brasil

**DOI:** 10.36660/abc.20260225

**Published:** 2026-09-25

**Authors:** Leonardo Miana, Bistra Zheleva, Juan Pedro Upegui Passos, Renata Mattos, Ana Paula Damiano, Jorge Yussef Afiune, Estela Suzana Kleiman Horowitz, Marcelo Biscegli Jatene

**Affiliations:** 1 Universidade de São Paulo São Paulo SP Brasil Universidade de São Paulo, São Paulo, SP – Brasil; 2 Children’s HeartLink Edina Minnesota EUA Children’s HeartLink, Edina, Minnesota – EUA; 3 Universidade Federal de Juiz de Fora Juiz de Fora MG Brasil Universidade Federal de Juiz de Fora, Juiz de Fora. MG – Brasil; 4 Instituto Nacional de Cardiologia Rio de Janeiro RJ Brasil Instituto Nacional de Cardiologia, Rio de Janeiro, RJ – Brasil; 5 Universidade Estadual de Campinas Campinas SP Brasil Universidade Estadual de Campinas, Campinas, SP – Brasil; 6 Instituto de Cardiologia do Distrito Federal Brasília DF Brasil Instituto de Cardiologia do Distrito Federal, Brasília, DF – Brasil; 7 Instituto de Cardiologia Fundação Universitária de Cardiologia Porto Alegre RS Brasil Instituto de Cardiologia / Fundação Universitária de Cardiologia (IC-FUC), Porto Alegre, RS – Brasil; 8 Hospital das Clínicas Faculdade de Medicina Universidade de São Paulo São Paulo SP Brasil Instituto do Coração do Hospital das Clínicas da Faculdade de Medicina da Universidade de São Paulo, São Paulo, SP – Brasil

**Keywords:** Cardiopatias Congênitas, Política de Saúde, Sistema Único de Saúde

## Abstract

**Fundamento:**

No Brasil, mais de 20.000 crianças nascem com cardiopatia congênita (CC) a cada ano, e uma proporção substancial necessita de tratamento cirúrgico ou intervencionista durante o primeiro ano de vida. Apesar dos avanços na assistência médica, persistem iniquidades significativas no acesso ao diagnóstico e ao tratamento em tempo oportuno.

**Objetivos:**

Descrever a evolução das políticas nacionais que orientam a atenção às CC no Brasil e discutir estratégias para aprimorar a organização e a prestação da assistência no sistema público de saúde do país.

**Métodos:**

Esta revisão narrativa e documental analisou dados de acesso público, políticas oficiais de saúde e literatura previamente publicada sobre a atenção às CC e a organização do sistema de saúde brasileiro.

**Resultados:**

Embora diversas iniciativas nacionais tenham sido implementadas ao longo do tempo, elas permanecem fragmentadas e não solucionaram plenamente as iniquidades no acesso à assistência. As principais lacunas incluem regionalização insuficiente, deficiências na coleta de dados, capacitação profissional inadequada e falta de coordenação do sistema de saúde.

**Conclusões:**

A atenção às CC no Brasil continua sendo caracterizada por iniquidades estruturais e políticas de saúde fragmentadas. O fortalecimento das redes regionalizadas de atenção, o aprimoramento das estratégias de financiamento e dos sistemas de dados e a otimização dos fluxos de encaminhamento são essenciais para garantir acesso oportuno e equitativo ao diagnóstico e ao tratamento.

## Graphical abstract

**Figura Central:**
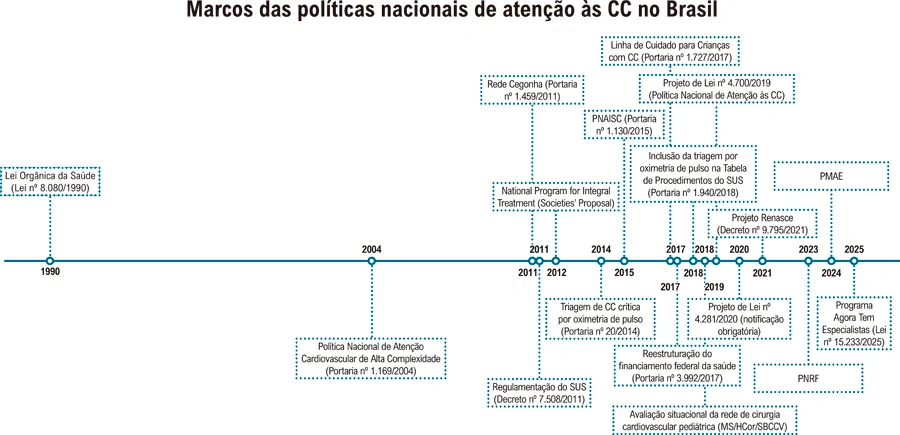
: Política Nacional de Atenção às Cardiopatias Congênitas: Reflexões e Propostas para o Fortalecimento da Assistência no Brasil

Política Nacional de Atenção às Cardiopatias Congênitas: Reflexões e Propostas para o Fortalecimento da Assistência no Brasil. Linha do tempo das políticas nacionais e dos marcos regulatórios para CC no Brasil (1990-2025). A figura apresenta cronologicamente os principais atos normativos, as iniciativas intersocietárias e os projetos de lei que estruturam o diagnóstico, o financiamento e o tratamento das CC no SUS. CC: cardiopatia congênita; HCor: Hospital do Coração; MS: Ministério da Saúde; PMAE: Programa Mais Acesso a Especialistas; PNAISC: Política Nacional de Atenção Integral à Saúde da Criança; PNRF: Programa Nacional de Redução das Filas; Renasce: Rede Nacional de Saúde Cardiovascular Especializada na Cardiopatia Congênita; SBCCV: Sociedade Brasileira de Cirurgia Cardiovascular; SUS: Sistema Único de Saúde.

## Introdução

A incidência global de cardiopatia congênita (CC) é estimada em 7-10 casos por 1.000 nascidos vivos.^1,2^ De acordo com os dados mais recentes do Instituto Brasileiro de Geografia e Estatística, aproximadamente 2,3 milhões de crianças nasceram no Brasil em 2023.^3^ Assim, estima-se que 20.000-23.000 crianças tenham nascido com CC,^4^ das quais pelo menos 50% necessitarão de tratamento cirúrgico ou transcateter ao longo da vida e 25% precisarão de intervenção durante o primeiro ano de vida.^5,6^

Consequentemente, aproximadamente 5.000 recém-nascidos e lactentes necessitam de um ou mais procedimentos cardíacos durante o primeiro ano de vida, enquanto outras 5.000 crianças precisam ser submetidas a procedimentos posteriormente. Considerando-se também as reintervenções, estima-se que sejam necessários anualmente no Brasil entre 12.000 e 14.000 procedimentos cirúrgicos e transcateter relacionados às CC.

Historicamente, apenas cerca de 7.000 procedimentos cirúrgicos relacionados às CC têm sido realizados anualmente no Sistema Único de Saúde (SUS). Como menos de 20% das crianças possuem planos privados de saúde, muitas crianças com CC podem viver e morrer sem receber a assistência médica necessária.^7,8^

Essa lacuna assistencial não é recente e tem sido documentada desde o início dos anos 2000, com índices particularmente preocupantes nas regiões Norte e Nordeste.^9^ Relatórios recentes e análises de sistemas de saúde em âmbito global indicam que uma proporção substancial das mortes relacionadas às CC na infância é evitável e que a ampliação do acesso oportuno a intervenções cirúrgicas ou transcateter de alta qualidade poderia reduzir a mortalidade em 58%.^10,11^ As evidências indicam que redes regionalizadas e centros especializados poderiam evitar uma proporção substancial dessas mortes.^10,11^ Apesar disso, o acesso à atenção às CC no Brasil permanece limitado, com acentuadas disparidades regionais e altas taxas de mortalidade após os procedimentos-índice.^12^ A regionalização também continua sendo um objetivo ainda não alcançado pelas políticas implementadas até o momento.^13^

Embora o SUS tenha sido fundamentado nos princípios da cobertura universal e da coordenação nacional, persistem lacunas históricas na implementação, no financiamento e na integração regional da atenção às CC. Este artigo revisa o desenvolvimento histórico das políticas públicas de atenção às CC no Brasil e analisa regulamentações e iniciativas relevantes.

## Métodos

Este estudo utilizou uma abordagem de análise de políticas de saúde fundamentada no modelo do triângulo de análise de políticas desenvolvido por Walt e Gilson em 1994.^14^ O modelo foi utilizado para avaliar sistematicamente a interação entre contexto, conteúdo, processos e atores envolvidos na evolução das políticas nacionais brasileiras de atenção às CC.

As fontes documentais foram identificadas por meio de busca sistemática em repositórios oficiais do governo, incluindo o Diário Oficial da União e a base de dados de atos normativos do Ministério da Saúde (MS) do Brasil. Os termos de busca incluíram “cardiopatia congênita”, “cirurgia cardiovascular pediátrica” e “triagem neonatal” . Foram considerados elegíveis leis federais, decretos e portarias publicados entre 1990 e 2025 que estabelecessem diretrizes para o financiamento da atenção às CC, o credenciamento de centros ou a organização de redes assistenciais no SUS, bem como projetos e documentos de posicionamento elaborados por sociedades profissionais e publicados em periódicos médicos.

Para avaliar as implicações práticas dessas políticas, a análise documental foi integrada a indicadores empíricos de saúde sintetizados por meio de uma revisão abrangente da literatura de estudos longitudinais. Esses indicadores foram avaliados ao longo do tempo e cotejados com as datas de implementação dos principais marcos das políticas, possibilitando uma análise crítica da associação entre as alterações legislativas e melhorias mensuráveis no acesso à assistência ou reduções das iniquidades regionais.

As políticas identificadas foram analisadas retrospectivamente para mapear a trajetória histórica da atenção às CC e identificar lacunas estruturais persistentes. Em seguida, foi desenvolvido um novo modelo para orientar a organização e a implementação de uma política nacional abrangente e equitativa para pessoas com CC no SUS. O modelo fundamentou-se na regionalização da assistência, na qualificação dos serviços e em uma regulação centralizada e eficiente.

O modelo proposto foi desenvolvido por meio de uma série de reuniões e discussões que envolveram representantes do MS e de quatro sociedades médicas: a Sociedade Brasileira de Cardiologia (SBC), representada por seu Departamento de Cardiopatias Congênitas e Cardiologia Pediátrica (DCC); a Sociedade Brasileira de Cirurgia Cardiovascular (SBCCV), representada por seu Departamento de Cirurgia Cardiovascular Pediátrica (DCCVPed); a Sociedade Brasileira de Hemodinâmica e Cardiologia Intervencionista (SBHCI), representada por seu Departamento de Intervenções em Cardiopatias Congênitas; e a Sociedade Brasileira de Pediatria (SBP), representada por integrantes de sua diretoria executiva. O modelo foi fundamentado na triangulação de três fontes de evidências: i) falhas de implementação identificadas em políticas anteriores; ii) indicadores empíricos da demanda não atendida por cirurgia cardíaca no Brasil; e iii) análise comparativa internacional de redes de atenção às CC com alto desempenho, adaptadas à estrutura federativa brasileira. Uma pessoa que ocupava o cargo de vice-presidente da Children’s HeartLink (CHL) participou de várias dessas discussões, tanto presencialmente quanto por meios virtuais, e forneceu orientação especializada durante todo o desenvolvimento do modelo.

## Resultados

### Histórico das iniciativas nacionais

#### Lei Orgânica da Saúde (Lei nº 8.080/1990) e desdobramentos posteriores

O SUS foi instituído pela Lei nº 8.080/1990 (Lei Orgânica da Saúde), que regulamentou o artigo 198 da Constituição Federal de 1988. Essa lei estabeleceu a responsabilidade do Estado de garantir acesso universal e equitativo à assistência à saúde, incluindo serviços diagnósticos e terapêuticos de alta complexidade para CC. O Decreto nº 7.508/2011 regulamentou adicionalmente a Lei nº 8.080/1990 ao definir a estrutura organizacional, os processos de planejamento em saúde e a articulação interfederativa do SUS. O decreto introduziu mecanismos fundamentais, incluindo as Regiões de Saúde, os Contratos Organizativos da Ação Pública da Saúde (COAPs) e o acesso regulado aos serviços de saúde, estabelecendo, assim, bases essenciais para redes de atenção destinadas a condições complexas como as CC.

## Políticas específicas de atenção cardiovascular, materna e infantil

### Portaria nº 1.169/GM/MS (2004)

Publicada em 15 de junho de 2004, a Portaria nº 1.169/GM/MS instituiu a Política Nacional de Atenção Cardiovascular de Alta Complexidade no SUS.^9,15^ A portaria estabeleceu critérios para a organização e o financiamento dos serviços públicos e contratualizados de saúde que realizavam procedimentos de média e alta complexidade. Sua principal contribuição foi a instituição formal de uma rede nacional e hierarquizada de atenção cardiovascular, estruturada de acordo com a regionalização, os ganhos de escala, o volume de procedimentos e a complexidade técnica.

Embora destinada a abranger a atenção cardiovascular em todas as faixas etárias, a portaria estabeleceu uma distinção relevante e pioneira ao criar uma categoria específica para cirurgias cardíacas estruturais congênitas e pediátricas e atribuir a esses procedimentos códigos técnicos diferentes daqueles utilizados nas operações cardíacas em pessoas adultas.^9,15^

Essa diferenciação resultou de um amplo processo de negociação e reconhecimento das necessidades específicas da cirurgia cardiovascular pediátrica. Ela precedeu o reconhecimento oficial da Cirurgia Cardiovascular Pediátrica e Congênita como subespecialidade médica pela Associação Médica Brasileira. Assim, já em 2004, o MS havia reconhecido a necessidade de uma abordagem diferenciada para a atenção às crianças com CC em razão dos desafios anatômicos, fisiológicos e organizacionais específicos envolvidos.

### Portaria nº 1.459/GM/MS — Rede Cegonha (2011)

Em 24 de junho de 2011, o MS instituiu a Rede Cegonha por meio da Portaria nº 1.459/GM/MS. Essa estratégia fortaleceu a atenção à saúde materna e infantil no SUS, com ênfase especial na assistência perinatal e no diagnóstico precoce de anomalias congênitas. Nesse contexto, foram incentivados a triagem neonatal e o diagnóstico de CC.

A estrutura da Rede Cegonha proporcionou as condições logísticas e clínicas necessárias ao diagnóstico precoce e à atenção coordenada aos recém-nascidos com condições graves, incluindo defeitos cardíacos congênitos. Também promoveu a vigilância neonatal e o registro sistemático de informações, ambos essenciais para garantir a rastreabilidade e a regulação da assistência aos recém-nascidos de alto risco.

Apesar de seu potencial, a Rede Cegonha não incorporou explicitamente os fluxos assistenciais para CC em seus protocolos oficiais, deixando uma lacuna entre os serviços de saúde materna e infantil e os serviços de cardiologia pediátrica de alta complexidade.

### Decreto nº 7.508/2011

O Decreto nº 7.508, publicado em 28 de junho de 2011, regulamentou a Lei nº 8.080/1990 e introduziu mecanismos fundamentais para a organização regional da prestação de serviços de saúde, incluindo as Regiões de Saúde, os Mapas da Saúde e os COAPs.

As seções a seguir descrevem como os princípios fundamentais estabelecidos por esse decreto podem influenciar diretamente a atenção às crianças com CC.

## Instituição das Regiões de Saúde

O decreto define as Regiões de Saúde como espaços geográficos nos quais as ações e os serviços de saúde são organizados de maneira integrada. Entretanto, a implementação efetiva da regionalização permanece um desafio persistente, resultando frequentemente na distribuição desigual de serviços e recursos. Persistem lacunas substanciais em diversas áreas do país, sobretudo no acesso à cirurgia cardiovascular pediátrica. Essas limitações ressaltam a necessidade de abordagens mais inovadoras e sensíveis ao contexto para adaptar os critérios de regionalização às diversas realidades brasileiras, tendo o acesso oportuno ao tratamento como consideração primordial.^13^

## Redes de Atenção à Saúde

O decreto introduziu as Redes de Atenção à Saúde como mecanismo de coordenação dos serviços entre diferentes níveis de complexidade.

## Contratos Organizativos da Ação Pública da Saúde

Os COAPs são instrumentos formais por meio dos quais os governos federal, estaduais e municipais definem suas respectivas responsabilidades. No contexto das CC, os COAPs poderiam ser utilizados para estabelecer metas regionais de cobertura cirúrgica; definir e formalizar as funções dos centros de excelência e dos hospitais regionais de apoio; e estabelecer mecanismos de regulação interestadual e financiamento proporcional.

## Mapa da Saúde

O decreto também determina que cada ente federativo elabore um Mapa da Saúde que identifique as necessidades de saúde da população e a disponibilidade dos serviços existentes.

## Programa Nacional para o Tratamento Integral de Crianças com Cardiopatia Congênita — 2012

Lançada em 2012 pela SBC, SBCCV, SBHCI e SBP, em colaboração com representantes do MS, essa iniciativa representou um dos esforços mais ambiciosos para desenvolver uma política pública abrangente e especializada de atenção às crianças com CC no Brasil.

Seu principal objetivo era assegurar acesso universal e equitativo à assistência diagnóstica, clínica, cirúrgica e intervencionista para crianças com CC por meio de um modelo federativo robusto, centrado na equidade regional e na qualidade da assistência. O programa propôs a criação da Secretaria Especial para o Tratamento de Crianças com Cardiopatia Congênita no MS, com autonomia técnica e financeira, bem como de uma rede nacional de Centros de Tratamento Integral da Cardiopatia Congênita, credenciados de acordo com diferentes níveis de complexidade. Também propôs a criação de uma Câmara Técnica Assessora (CTA) nacional, composta por especialistas médicos e representantes da sociedade civil.

Apesar de seu rigor técnico e apoio institucional, o programa nunca foi implementado em razão de diversas barreiras.

### Portaria nº 20/SCTIE/MS — 10 de junho de 2014

Essa portaria incorporou formalmente a triagem de CC crítica por oximetria de pulso, popularmente conhecida no Brasil como “teste do coraçãozinho”, à triagem neonatal universal no SUS. A decisão seguiu uma recomendação da Comissão Nacional de Incorporação de Tecnologias no SUS, que reconheceu a importância da oximetria de pulso para a detecção precoce de CC em recém-nascidos.

### Portaria nº 1.130/GM/MS — Política Nacional de Atenção Integral à Saúde da Criança (2015)

A Portaria nº 1.130, publicada em 5 de agosto de 2015, instituiu a Política Nacional de Atenção Integral à Saúde da Criança (PNAISC) no SUS. Essa política representou um marco importante ao alinhar os princípios da atenção integral, da colaboração intersetorial, da equidade e da participação social às necessidades de saúde em constante evolução das crianças de 0-9 anos no Brasil.

Embora não fosse especificamente direcionada às CC, a PNAISC criou oportunidades importantes para fortalecer a organização da assistência às crianças com doenças cardíacas.

### Portaria nº 1.727/GM/MS (2017)

A Portaria nº 1.727, publicada em 11 de julho de 2017, instituiu a Linha de Cuidado para Crianças com Cardiopatia Congênita no SUS. Foi a primeira regulamentação federal elaborada especificamente para sistematizar e integrar a atenção às crianças com CC em todo o país. Seu principal objetivo era estabelecer diretrizes e fluxos assistenciais que garantissem acesso contínuo, oportuno e de qualidade ao diagnóstico, ao tratamento cirúrgico ou intervencionista e ao acompanhamento ambulatorial, em conformidade com os princípios de regionalização, equidade e integralidade do SUS.

A implementação da Portaria nº 1.727 foi apoiada pela Portaria nº 3.992/GM/MS (2017), que reestruturou o financiamento federal da saúde no SUS. Essa regulamentação complementar proporcionou mecanismos financeiros essenciais para a Linha de Cuidado ao padronizar a transferência de recursos para estados e municípios.

### Portaria nº 1.940 — 28 de junho de 2018

Essa portaria incorporou formalmente a triagem de CC crítica por oximetria de pulso à Tabela de Procedimentos do SUS e atribuiu-lhe um código específico. Essa medida possibilitou sua implementação rotineira e seu reembolso como parte do programa nacional de triagem neonatal.

## Avaliação situacional da rede de cirurgia cardiovascular pediátrica no Sistema Único de Saúde

Esse projeto foi conduzido por meio de uma parceria entre o MS, o Hospital do Coração (HCor) e o DCCVPed, com financiamento do Programa de Apoio ao Desenvolvimento Institucional do SUS.

A iniciativa buscou realizar uma avaliação técnica, quantitativa e qualitativa de todos os centros de cirurgia cardíaca pediátrica credenciados ou em atividade para subsidiar políticas públicas e decisões estratégicas relacionadas à reorganização da rede. Seus métodos incluíram visitas técnicas presenciais, entrevistas com equipes locais, formulários padronizados para coleta de dados, auditorias documentais e análise de dados do Departamento de Informática do SUS (DATASUS). Dos 69 centros credenciados até o final de 2018, dois haviam encerrado suas atividades e 13 se recusaram a participar, dos quais 12 não realizavam procedimentos cirúrgicos. Consequentemente, foram avaliados, em 2019, 44 centros públicos e filantrópicos de cirurgia cardíaca pediátrica distribuídos por 22 estados brasileiros.

Entretanto, o projeto apresentou limitações importantes. Nenhum relatório nacional consolidado foi oficialmente publicado, embora relatórios individuais tenham sido fornecidos aos centros participantes e um artigo científico de autoria de integrantes do DCCVPed tenha apresentado resultados parciais.^7^ Além disso, o acompanhamento institucional limitado e a utilização insuficiente dos dados obtidos pelo MS reduziram o impacto do projeto sobre as políticas públicas em curto prazo.

### Projeto de Lei nº 4.700/2019 (alterado em 2023)

Apresentado no Congresso Nacional, o Projeto de Lei nº 4.700/2019 propõe a instituição de uma política nacional de atenção às CC, com diretrizes para diagnóstico precoce, tratamento oportuno e acompanhamento longitudinal. Uma alteração apresentada em 2023 estabeleceu objetivos mais claros relacionados à regionalização, ao financiamento, à formação da força de trabalho e ao acesso equitativo. O projeto de lei permanece em tramitação legislativa, sem cronograma de implementação aprovado.

### Projeto de Lei nº 4.281/2020

Esse projeto de lei exigiria que os recém-nascidos diagnosticados com CC por meio da triagem por oximetria de pulso fossem notificados aos serviços especializados. A medida proposta tem como objetivo facilitar o encaminhamento precoce e o acompanhamento adequado. Esperava-se que o Poder Executivo publicasse a regulamentação necessária no prazo de 120 dias, mas essa regulamentação ainda não foi publicada.

### Decreto nº 9.795/2021 — Projeto Rede Nacional de Atenção à Saúde da Criança com Cardiopatia Congênita

Em 17 de maio de 2021, o Decreto nº 9.795 instituiu o Projeto Rede Nacional de Saúde Cardiovascular Especializada na Cardiopatia Congênita (Renasce). O projeto buscou reorganizar a rede nacional de atenção, com ênfase na regionalização, na ampliação da capacidade dos serviços, na capacitação da força de trabalho, na telemedicina e na regulação centralizada da assistência cirúrgica e clínica.

O projeto estabeleceu objetivos ambiciosos, incluindo a redução da mortalidade relacionada às CC mediante a garantia de acesso oportuno à cirurgia e à assistência especializada, a organização dos centros de acordo com os níveis de complexidade, a criação de uma CTA e a implementação de uma lista nacional de espera unificada.

Apesar das barreiras à implementação, o Projeto Renasce representou um passo importante rumo a uma política nacional de atenção às CC sustentável, tecnicamente robusta e coordenada regionalmente. Suas estruturas metodológicas e conclusões diagnósticas permanecem relevantes e fornecem uma base sólida para a retomada dos esforços estratégicos em 2026.

### Lei nº 15.233/2025 — Agora Tem Especialistas

O Programa Agora Tem Especialistas, instituído pela Lei nº 15.233 e pela Portaria GM/MS nº 7.266/2025 do MS, representa o desenvolvimento mais recente dessa trajetória política. A iniciativa surgiu em 2023 como o Programa Nacional de Redução das Filas, concentrado em procedimentos cirúrgicos eletivos. Em 2024, evoluiu para o Programa Mais Acesso a Especialistas, ampliando seu escopo para incluir serviços ambulatoriais especializados e diagnósticos. Em 2025, a iniciativa foi consolidada e novamente ampliada, passando a abranger assistência ambulatorial especializada, cirurgias eletivas, radioterapia, incentivos financeiros, iniciativas de saúde digital no SUS, mecanismos de ressarcimento, provimento e capacitação da força de trabalho e serviços especializados complementares.

Embora a cardiologia seja identificada como área prioritária, possibilitando a ampliação do acesso a consultas, exames diagnósticos e procedimentos eletivos, a cardiologia pediátrica e as CC não são abordadas especificamente. Para incorporar efetivamente a cardiologia pediátrica e congênita, os componentes do programa devem ser alinhados aos fluxos de atenção às CC. Esse alinhamento deve promover o acesso oportuno à avaliação ambulatorial especializada e à ecocardiografia, fortalecer a regulação baseada em risco e o encaminhamento para intervenções que não podem ser adiadas e utilizar ferramentas de saúde digital para apoiar a continuidade da assistência e o monitoramento dos desfechos.

Nas últimas 2 décadas, a Linha de Cuidado para Crianças com Cardiopatia Congênita evoluiu gradualmente, com diversas iniciativas convergindo para um modelo horizontalmente integrado que se estende do diagnóstico pré-natal ao acompanhamento em longo prazo. Esses esforços priorizaram a identificação de centros de referência, a capacitação de equipes especializadas e o desenvolvimento de centros regionais para garantir acesso universal e equitativo à assistência padronizada. Entretanto, permanece urgente a necessidade de um banco de dados nacional robusto e centralizado que forneça informações epidemiológicas e clínicas confiáveis sobre anomalias congênitas, vigilância das CC e desfechos de morbidade e mortalidade. Esses dados são essenciais para orientar as políticas públicas, mas o DATASUS tem sido historicamente insuficiente para essa finalidade. A integração formal da telemedicina à rede assistencial é igualmente importante para promover o acesso equitativo e fortalecer a capacidade regional.

A Figura Central oferece mais do que uma visão cronológica: ela demonstra a lacuna de implementação existente na rede brasileira de atenção às CC. Quando a linha do tempo é examinada em conjunto com os indicadores apresentados na Figura 1, observa-se que marcos regulatórios importantes, como a Linha de Cuidado de 2017, aparentemente não produziram mudança discernível no déficit persistente de tratamento cirúrgico relatado por Caneo et al.^7^

**Figura 1:**
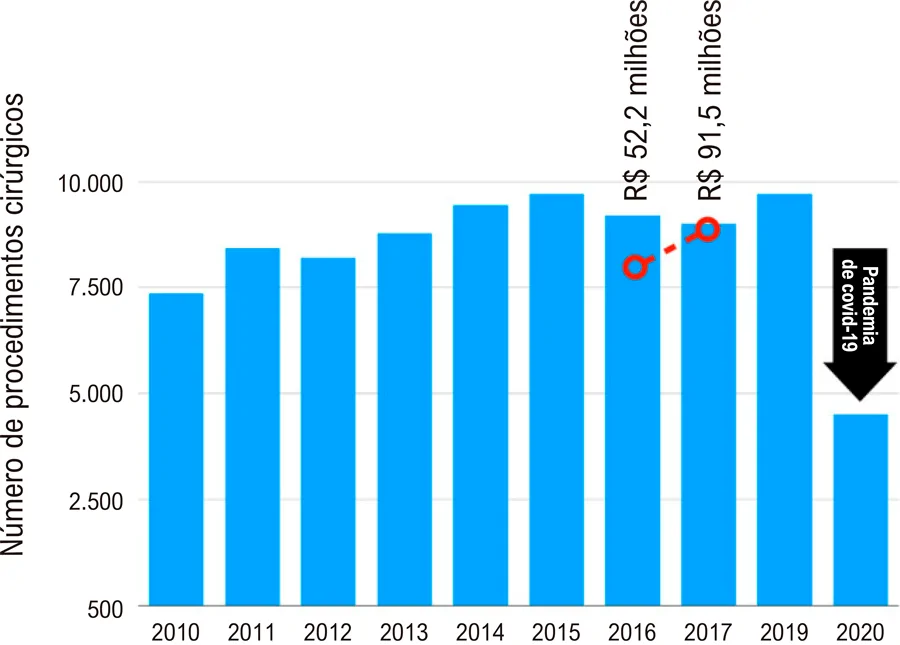
– Número de procedimentos cirúrgicos cardíacos pediátricos realizados no Brasil, 2010-2020. Adaptado de Caneo et al.7

Número de procedimentos cirúrgicos

Essa comparação visual sustenta a interpretação de que persistem lacunas substanciais de implementação: o número crescente de portarias contrasta acentuadamente com a melhora limitada dos desfechos clínicos. Essa dissociação sugere que a atual estrutura política atingiu um limite estrutural e que a inclusão de novos dispositivos normativos sem a correspondente reestruturação operacional provavelmente não melhorará o acesso à assistência.

## Impacto das políticas sobre os indicadores assistenciais

A análise longitudinal revelou uma lacuna persistente entre o desenvolvimento e a implementação das políticas. Apesar da instituição da Política Nacional de Atenção Cardiovascular de Alta Complexidade em 2004 e da Linha de Cuidado em 2017, o déficit nacional de tratamento cirúrgico permaneceu criticamente elevado.^14,16^ Segundo Barreto et al.,^17^ o déficit de cirurgias corretivas era de 64,27% em 2010 e diminuiu apenas modestamente para 51,46% em 2019, antes de voltar a aumentar durante a pandemia de covid-19 (Figura 2). As disparidades regionais permaneceram uma importante barreira estrutural: a Região Norte apresentou consistentemente o maior déficit acumulado (75,87%), em comparação com a Região Sul (47,32%).^12,17^ Além disso, embora a obrigatoriedade da triagem por oximetria de pulso em recém-nascidos tenha melhorado o diagnóstico precoce, ela não foi acompanhada por uma expansão proporcional do número de centros cirúrgicos plenamente operacionais, conforme demonstrado pela avaliação situacional de 2019.^7^

**Figura 2:**
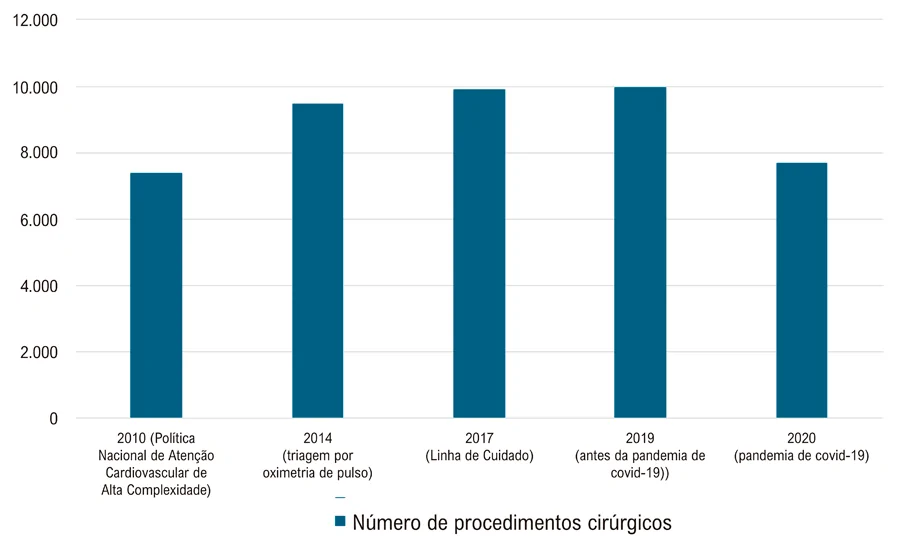
– Tendências do volume cirúrgico para CC no Brasil. Adaptado de Barreto et al.17 O gráfico mostra o aumento progressivo do número absoluto de procedimentos cirúrgicos relacionados às CC, que atingiu o pico de 9.956 em 2019, antes da pandemia de covid-19. CC: cardiopatia congênita.

Em relação às tendências de mortalidade, Cohen et al.^18^ identificaram uma mudança significativa a partir de 2018, 3 anos após a triagem por oximetria de pulso em recém-nascidos ter se tornado obrigatória. Entretanto, essa redução não foi uniforme. Embora a taxa nacional de mortalidade tenha diminuído, a Região Norte apresentou comportamento discrepante, com aumento da mortalidade durante o mesmo período.^18^

Esses achados demonstram uma dissociação substancial entre o avanço regulatório e os desfechos clínicos no Brasil. Embora políticas como a Portaria nº 1.727/2017 fossem tecnicamente robustas, sua implementação enfrentou barreiras críticas. Conforme destacado por Soares et al.^16^ o objetivo de ampliar a capacidade assistencial em 30% ao ano permanece amplamente não alcançado em razão do subfinanciamento crônico e da escassez de profissionais de saúde especializados.^16^ A transição para o financiamento por orçamento global estabelecida pela Portaria nº 3.992/2017 frequentemente diluiu os recursos destinados à atenção às CC, o que ajuda a explicar por que a regionalização permaneceu, em grande medida, um objetivo político, em vez de uma realidade operacional. A ausência de um banco de dados clínicos unificado que vá além dos dados administrativos de faturamento disponibilizados pelo DATASUS continua sendo uma barreira importante à identificação de pontos específicos de falha ao longo da Linha de Cuidado e impede ajustes da estratégia nacional orientados por desfechos.

A comparação entre os marcos legislativos e os indicadores de prestação da assistência revelou uma lacuna substancial de implementação na rede brasileira de atenção às CC. Conforme mostrado na Figura 2, embora o número absoluto de procedimentos cirúrgicos tenha aumentado progressivamente e atingido o pico de 9.956 em 2019, esse crescimento foi insuficiente para superar o déficit estrutural. Os dados sintetizados de Barreto et al.^17^ e Miana et al.^12^ mostram que o déficit nacional de tratamento cirúrgico permaneceu acima de 50% durante a última década (Figura 3). Esses achados sugerem que o sistema atingiu um platô estrutural, apesar dos avanços regulatórios introduzidos pela Linha de Cuidado de 2017.

**Figura 3:**
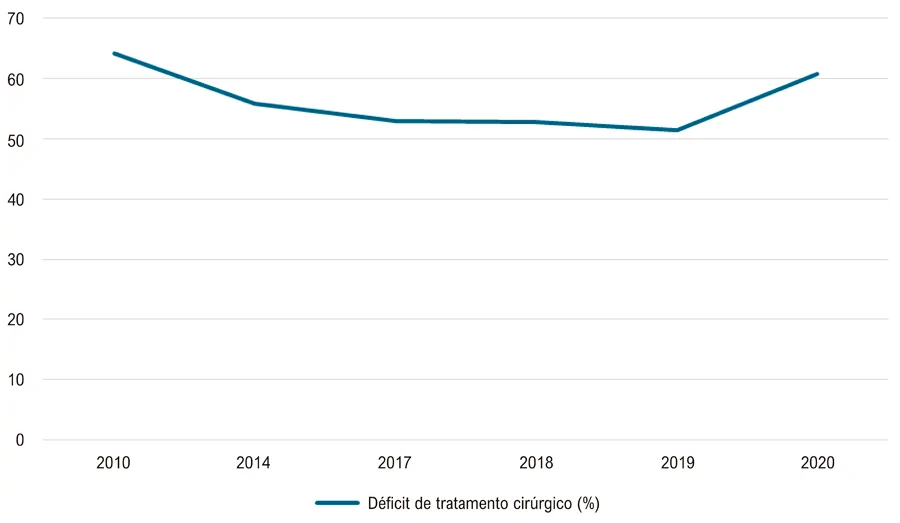
– Tendências longitudinais do déficit nacional de tratamento cirúrgico e da mortalidade relacionada às CC no Brasil. Adaptado de Barreto et al.17 O déficit nacional de tratamento cirúrgico permaneceu consistentemente acima de 50%, indicando um platô estrutural da capacidade assistencial. CC: cardiopatia congênita.

## Proposta de novo modelo

A necessidade de um banco de dados nacional de CC robusto e centralizado é ressaltada pelas limitações inerentes aos sistemas administrativos de informação existentes. Conforme demonstrado por Miana et al.,^12^ o Sistema de Informações Hospitalares do SUS não apresenta granularidade clínica suficiente para distinguir diferentes níveis de complexidade anatômica ou acompanhar desfechos funcionais em longo prazo. Barreto et al.^17^ também observaram que a dependência de dados secundários de faturamento pode resultar em subnotificação e inconsistências de codificação, ocultando, assim, a verdadeira magnitude do déficit de tratamento cirúrgico. A ausência de um registro clínico comparável ao Banco de Dados de Cirurgia Cardíaca Congênita da Society of Thoracic Surgeons (STS-CHSD), da América do Norte, impede a implementação do financiamento baseado em desfechos e do monitoramento da qualidade ajustado ao risco, ambos componentes habituais das redes de atenção às CC com alto desempenho.

O modelo proposto é uma estrutura baseada em evidências, desenvolvida por meio da triangulação de três pilares analíticos: i) identificação das falhas de implementação no Brasil; ii) análise comparativa internacional de redes com alto desempenho, como o STS-CHSD; e iii) consenso entre representantes do MS, SBC, SBCCV, SBHCI e SBP, com orientação da CHL. O modelo substitui uma abordagem predominantemente centrada na regulamentação por uma estratégia orientada à implementação.

Para viabilizar essa transformação operacional, o modelo está organizado em torno dos seguintes pilares estratégicos:

1. Regionalização da atenção às CC por meio de redes macrorregionais integradas e vinculadas a centros de excelência.
2. Implementação de programas de educação continuada e capacitação remota para as equipes dos hospitais regionais, apoiados por protocolos compartilhados e teleconsultorias.
3. Reestruturação da Central Nacional de Regulação de Alta Complexidade (CNRAC) para CC, a fim de melhorar a eficiência e a transparência dos processos de encaminhamento e transporte de pacientes.
4. Estabelecimento de um modelo regulatório que combine planejamento federal e implementação regional descentralizada.
5. Monitoramento em tempo real dos desfechos e indicadores de qualidade por meio de um banco de dados clínicos específico, independente do DATASUS, para avaliar indicadores de saúde relacionados às CC.
6. Otimização da alocação e da utilização de recursos ao longo de toda a linha de atenção às CC no SUS.

Recomendamos que o MS lidere um processo nacional de planejamento consultivo, com a participação dos atores relevantes, para estabelecer padrões, definir prioridades e elaborar um roteiro para a atenção cardiológica pediátrica e congênita no Brasil. Esse processo deve orientar uma estratégia nacional que aborde a formação da força de trabalho, a infraestrutura, a capacidade dos serviços e o financiamento. As iniciativas subsequentes devem fortalecer a triagem, o encaminhamento e a assistência clínica, cirúrgica e intervencionista às crianças com CC. Essas iniciativas devem estar alinhadas às prioridades estratégicas nacionais e contribuir para o cumprimento dos Objetivos de Desenvolvimento Sustentável relacionados à redução de mortes evitáveis entre recém-nascidos e crianças.

### 1. Regionalização da assistência

A primeira etapa envolve mapear a capacidade assistencial de cada macrorregião, definir os fluxos de referência e contrarreferência entre as unidades locais e os centros de excelência e estabelecer metas regionais e nacionais de prestação de serviços.

### 2. Parcerias com centros de excelência

Os Centros Nacionais de Excelência serão selecionados de acordo com critérios técnicos objetivos, incluindo volume de casos, desfechos clínicos, composição das equipes multidisciplinares, atuação acadêmica e localização estratégica. Esses centros estabelecerão parcerias para apoiar a educação profissional continuada, a supervisão técnica e as atividades regulatórias.

O sistema atual distingue Unidades Assistenciais de Centros de Referência. No modelo proposto, essas categorias serão redefinidas de acordo com critérios de desempenho e qualidade, com a manutenção ou revisão de suas denominações, conforme apropriado. Também será criado um núcleo técnico nacional de coordenação, com representantes das Unidades Assistenciais e dos Centros de Referência.

### 3. Educação continuada e capacitação remota

Com base nas iniciativas existentes do MS, incluindo aquelas implementadas no Instituto do Coração da Universidade de São Paulo e no HCor, será desenvolvido um programa de capacitação contínua que combinará atividades presenciais e remotas e abrangerá todas as etapas da atenção às CC. O programa também apoiará profissionais da atenção primária no reconhecimento das CC e na coordenação da assistência às crianças afetadas.

### 4. Reestruturação da regulação e da transferência de pacientes

Embora já existam mecanismos como a CNRAC e as Centrais Regionais de Regulação de Alta Complexidade, o modelo proposto prevê a reformulação dos processos de triagem e autorização. Os processos regulatórios serão totalmente digitalizados e orientados por critérios clínicos objetivos e prazos máximos de resposta. Um sistema de priorização considerará tanto o risco clínico quanto a acessibilidade geográfica. Pacientes em estado crítico terão acesso a transporte aeromédico por meio de parcerias público-privadas.

### 5. Governança federal com execução regional

O desenvolvimento das políticas, o financiamento e a avaliação de desempenho permanecerão centralizados no MS, enquanto a implementação operacional ocorrerá por meio de redes regionais coordenadas.

Será instituída uma CTA Nacional com atribuições consultivas e deliberativas. Para assegurar a coordenação entre todos os níveis de atenção, também será criado um Comitê Gestor Nacional de CC, de acordo com a seguinte estrutura de governança:

- Liderança estratégica: o MS manterá a responsabilidade principal pelo financiamento, pela regulação nacional por meio da CNRAC e pela supervisão das políticas.
- Governança técnica: uma CTA Nacional permanente, composta por representantes da SBC, SBCCV, SBHCI e SBP, estabelecerá protocolos clínicos e padrões de credenciamento.
- Avaliação independente: a CHL atuará como parceira técnica internacional, apoiando a certificação independente dos Centros de Excelência de acordo com parâmetros globais de desempenho.

### 6. Acesso aos dados e monitoramento

Melhorar a acessibilidade e a integração dos dados é essencial para acompanhar pacientes ao longo de suas trajetórias assistenciais no SUS. A partir de 2026, o diagnóstico de CC de cada paciente será vinculado ao respectivo número de inscrição no Cadastro de Pessoas Físicas, possibilitando o acompanhamento longitudinal de internações, procedimentos, tratamentos clínicos e desfechos. Essa abordagem favorecerá a vigilância em tempo real, o planejamento orientado por dados e o desenvolvimento de um banco de dados epidemiológicos nacional robusto. A necessidade de um banco de dados nacional centralizado decorre da quantidade limitada de informações clínicas disponíveis nos sistemas existentes.

### 7. Aspectos específicos da contrarreferência e da regulação

A organização efetiva da atenção às CC exige não apenas a identificação dos centros de referência e a centralização dos processos regulatórios, mas também a formalização dos fluxos de contrarreferência e o estabelecimento de salvaguardas éticas que garantam acesso equitativo e transparente.

Quando um paciente for considerado inelegível para transferência ou cirurgia, será realizada uma teleconsulta obrigatória com sua família. Durante essa consulta, a justificativa clínica para a decisão será explicada e formalmente documentada. Após a estabilização clínica, os pacientes retornarão às unidades de origem por meio de um fluxo formal de contrarreferência, a fim de garantir a continuidade da assistência e evitar sobrecarga desnecessária dos centros terciários.

### 8. Parcerias e cooperação internacionais

A colaboração técnica com a CHL poderá apoiar a seleção, a capacitação e a certificação dos Centros de Excelência e dos Centros Regionais de Capacitação e Educação, de acordo com padrões internacionais de qualidade, sustentabilidade e impacto.

### 9. Resultados esperados

- Redução das listas de espera para procedimentos cirúrgicos relacionados às CC.
- Redução da mortalidade infantil atribuível a doenças cardíacas tratáveis.
- Expansão da rede de centros regionais credenciados e qualificados.
- Ampliação da capacidade regional e redução da necessidade de transferência de pacientes.
- Aprimoramento da tomada de decisões clínicas e regulatórias.
- Melhoria dos indicadores de desfechos e maior abrangência dos dados de acompanhamento em longo prazo.

### 10. Atores e parceiros propostos

- MS, por meio do Departamento de Atenção Especializada e Temática e da Secretaria de Atenção Especializada à Saúde.
- Conselho Nacional de Secretários de Saúde.
- Conselho Nacional de Secretarias Municipais de Saúde.
- Programas credenciados de cirurgia cardíaca congênita.
- SBC, por meio do DCC.
- SBCCV, por meio do DCCVPed.
- SBHCI.
- SBP.
- CHL.

### 11. Cronograma de implementação

**Fase 1: planejamento e estruturação organizacional (até dezembro de 2027)**

- Definir os Centros Nacionais de Excelência de acordo com critérios técnicos objetivos.
- Selecionar e credenciar os Centros Regionais de Capacitação e Educação.
- Estabelecer acordos com as secretarias estaduais e municipais de saúde sobre os fluxos de encaminhamento e os Centros de Referência.
- Capacitar os serviços regionais para cadastrar pacientes na lista nacional de espera regulada pela CNRAC.

**Metas até o final de 2027:**

- Todos os Centros de Referência identificados e credenciados.
- Todos os serviços participantes qualificados para cadastrar pacientes na plataforma nacional.

**Fase 2: implementação progressiva e regulação centralizada (até 2029)**

1. Implementar o Sistema Nacional Unificado de Regulação sob a coordenação da CNRAC.
2. Implementar um indicador nacional de priorização baseado no tempo de espera e na complexidade dos casos.
3. Redirecionar automaticamente os casos urgentes que não forem aceitos por um centro no prazo de 48 horas.
4. Definir a capacidade de cada centro de acordo com seu perfil de complexidade.
5. Exigir teleconsulta para pacientes considerados inelegíveis para transferência ou cirurgia.
6. Exigir contrarreferência após a estabilização clínica.

**Metas até o final de 2029:**

- Implementação do programa em todo o território nacional.
- Resolução de 80% das solicitações da CNRAC no prazo de 48 horas.
- Sistema regulatório plenamente operacional, transparente e tecnicamente orientado.
- Fluxos funcionais de contrarreferência em todas as regiões.
