# Supplementary material for: Política Nacional de Atenção às Cardiopatias Congênitas: Reflexões e Propostas para o Fortalecimento da Assistência no Brasil
Source: Arq Bras Cardiol. 2026 Sep 25;123(8):e20260225. [Article in Portuguese] doi: 10.36660/abc.20260225 (PMC13713000; doi:10.36660/abc.20260225)
Supplement: Translation

# Translation: National Policy on Congenital Heart Disease: Reflections and Proposals for Strengthening Care in Brazil

**DOI:** 10.36660/abc.20260225i

**Authors:** Leonardo Miana, Bistra Zheleva, Juan Pedro Upegui Passos, Renata Mattos, Ana Paula Damiano, Jorge Yussef Afiune, Estela Suzana Kleiman Horowitz, Marcelo Biscegli Jatene

**Affiliations:** 1 Brazil Universidade de São Paulo, São Paulo, SP – Brazil; 2 USA Children’s HeartLink, Edina, Minnesota – USA; 3 Brazil Universidade Federal de Juiz de Fora, Juiz de Fora. MG – Brazil; 4 Brazil Instituto Nacional de Cardiologia, Rio de Janeiro, RJ – Brazil; 5 Brazil Universidade Estadual de Campinas, Campinas, SP – Brazil; 6 Brazil Instituto de Cardiologia do Distrito Federal, Brasília, DF – Brazil; 7 Brazil Instituto de Cardiologia / Fundação Universitária de Cardiologia (IC-FUC), Porto Alegre, RS – Brazil; 8 Brazil Instituto do Coração do Hospital das Clínicas da Faculdade de Medicina da Universidade de São Paulo, São Paulo, SP – Brazil

**Keywords:** Congenital Heart Defect, Health Policy, Unified Health System

## Abstract

**Background:**

In Brazil, more than 20,000 children are born annually with congenital heart disease, and a significant proportion require surgical or interventional treatment within the first year of life. Despite advances in medical care, substantial inequalities persist in access to timely diagnosis and treatment.

**Objectives:**

To describe the evolution of national policies related to congenital heart disease care in Brazil and to discuss strategies for improving the organization and delivery of care within the national public health system.

**Methods:**

This is a narrative and documental review based on the analysis of publicly available data, official health policies, and previously published literature addressing congenital heart disease care and health system organization in Brazil.

**Results:**

The analysis identified that although multiple national initiatives have been implemented over time, they remain fragmented and have not fully addressed disparities in access to care. Key gaps include deficiencies in regionalization, data collection, workforce training, and system coordination.

**Conclusions:**

Congenital heart disease care in Brazil remains marked by structural inequalities and fragmentation of health policies. Strengthening regionalized networks, improving financing strategies, enhancing data systems, and optimizing referral pathways are essential to ensure timely and equitable access to diagnosis and treatment.

## Introdution

The global incidence of congenital heart diseases (CHDs) is estimated at 7 to 10 per 1,000 live births.^1,2^ In Brazil, according to the most recent data from the Brazilian Institute of Geography and Statistics (IBGE), approximately 2.3 million children were born in 2023.^3^ Thus, an estimated 20,000 to 23,000 were born with CHDs,^4^ of whom at least 50% will require surgical or percutaneous treatment during their lifetime, and one quarter need intervention within the first year of life.^5,6^ Consequently, each year, approximately 5,000 newborns and infants require one or more cardiac surgeries in their first year, in addition to another 5,000 children needing procedures later in life, plus the number of patients that need reinterventions. It is then estimated that between 12,000 and 14,000 CHD-related operations and percutaneous interventions are necessary annually in Brazil.

Historically, however, only around 7,000 CHD surgeries are performed annually within Brazil’s Unified Health System (SUS), and considering that less than 20% of children are covered by private health insurance, it can be inferred that a large proportion of children with CHD live and die without receiving the medical care they need.^7,8^

This deficit is not new, having been highlighted since the early 2000s, with alarming rates, especially in the North and Northeast regions.^9^ Reports and recent global health-system analyses indicate that a substantial proportion of CHD-related childhood deaths is preventable, and that improving access to timely, high-quality surgery and/or percutaneous intervention may reduce mortality by 58%.^10,11^ Data have shown that regionalized networks and specialized centers can avert a substantial share of preventable mortality.^10,11^ In contrast, Brazil still faces limited access, marked by striking regional inequalities and high mortality rates after index surgeries,^12^ with regionalization remaining a persistent and often unrealized challenge in implemented policies.^13^

Despite the Brazilian SUS ideal foundation for universal coverage and national coordination, historical gaps in CHD care implementation, funding, and regional integration persist. This article reviews the historical development of public policies for CHDs in Brazil, analyzing regulations and initiatives.

## Methods

This study employs a Health Policy Analysis approach, grounded in the policy analysis triangle framework developed by Walt and Gilson in 1994.^14^ This framework was used to systematically evaluate the interplay between the context, content, process, and actors involved in the evolution of Brazil’s national policies for CHD.

Documentary sources were identified through a systematic search of official government repositories, such as the Diário Oficial da União (DOU) and the Ministry of Health’s normative database. Search strategies utilized descriptors such as “congenital heart disease,” “pediatric cardiovascular surgery,” and “neonatal screening.” Inclusion criteria focused on federal laws, decrees, and ordinances published between 1990 and 2025 that established guidelines for CHD financing, center accreditation, or care networks within the Unified Health System (SUS), as well as projects and position papers from professional societies published in medical journals.

To assess the practical implications of these policies, the documentary analysis was integrated with empirical health indicators synthesized from a comprehensive literature review of longitudinal studies.

These indicators were analyzed longitudinally and cross-referenced with the implementation dates of major policy milestones. This allowed for a critical evaluation of whether legislative changes were associated with measurable improvements in access to care or a reduction in regional inequities.

The identified policies were analyzed retrospectively to map the historical trajectory of CHD care and identify persistent structural gaps. A new model was then described to structure and implement a comprehensive and equitable national policy for the care of patients with CHD within Brazil’s SUS, grounded in regionalized care, service qualification, and centralized, efficient regulation. The proposed new model was constructed after a series of encounters and discussions between MoH representatives and four medical societies representatives: the Brazilian Society of Cardiology (SBC), represented by its Department of Pediatric Cardiology (DCC); the Brazilian Society of Cardiovascular Surgery (SBCCV), represented by its Department of Pediatric and Congenital Cardiovascular Surgery (DCCVPed); the Brazilian Society of Hemodynamics and Interventional Cardiology (SBHCI), represented by its Department of Congenital Heart Interventions (DICC); and the Brazilian Society of Pediatrics (SBP), represented by its leadership board, using a triangulation of evidence, combining: (1) the identification of implementation failures in previous policies; (2) empirical indicators of unmet surgical demand in Brazil; and (3) international benchmarking of high-performance CHD networks, adapted to the Brazilian federative context, plus counselling from Children’s Heartlink vice-president that took part in many of these discussions both live and online.

## Results

### Historical Background of National Initiatives

#### Organic Health Law (Law No. 8,080/1990) and its developments

Brazil’s Unified Health System (SUS) was established by Law No. 8,080/1990 (the Organic Health Law), which implemented Article 198 of the 1988 Constitution. This law enshrined the State’s duty to ensure universal and equitable access to healthcare, including high-complexity CHD diagnosis and treatment. Decree No. 7,508/2011 further regulated Law No. 8,080, defining SUS’s organizational structure, health planning, and inter-federative coordination. It introduced key concepts such as Health Regions, Organizational Contracts for Public Health Action (COAP), and regulated access, forming essential frameworks for care networks, including those for complex conditions such as CHD.

## Specific policies for cardiovascular, maternal, and child care

### Ordinance No. 1,169/GM/MS (2004)

Published on June 15, 2004, Ordinance No. 1,169/GM/MS marked the creation of the *National Policy for High-Complexity Cardiovascular Care* within SUS.^9,15^ This ordinance established criteria for organizing and financing public and contracted services responsible for medium and high-complexity cardiac procedures. Its main contribution was the formal establishment of a nationwide, hierarchical cardiovascular care network based on regionalization, scale, procedural volume, and technical complexity.

Although designed to encompass cardiovascular care across all age groups, the ordinance made a notable and pioneering distinction by specifically and separately defining congenital and pediatric structural cardiac surgery procedures, assigning unique technical codes distinct from those of adult cardiac operations.^9,15^

This differentiation was fundamental, being the result of an intense process of negotiation and recognition of the specificities of pediatric cardiovascular surgery. This recognition, issued well before Pediatric and Congenital Cardiovascular Surgery was officially acknowledged as a medical subspecialty by the Brazilian Medical Association (AMB), demonstrates that, as early as 2004, the Ministry of Health had already recognized the need for a differentiated approach for this population, given the unique anatomical, physiological, and organizational challenges involved in the management of CHD.

### Ordinance No. 1,459/GM/MS – Rede Cegonha (2011)

On June 24, 2011, the MoH launched the Rede Cegonha (*Stork Network*) through Ordinance No. 1,459. This strategy strengthened maternal and child healthcare within the SUS, with a particular focus on perinatal care and early diagnosis of congenital anomalies. Within this framework, the inclusion of screening and diagnosis of CHD in the neonatal period was encouraged.

The Rede Cegonha framework provided both the logistical and clinical conditions necessary for early diagnosis and coordinated care of newborns with severe conditions, including congenital heart defects. Furthermore, it promoted a culture of neonatal surveillance and record-keeping, essential for traceability and regulatory management of high-risk cases in the neonatal period.

Despite its potential, the Rede Cegonha did not explicitly incorporate CHD pathways into its official protocols, creating a gap between maternal/child care and high-complexity pediatric cardiology services.

### Decree No. 7,508/2011

Decree No. 7,508, issued on June 28, 2011, regulated Law No. 8,080/1990 and introduced key mechanisms for the regional organization of healthcare delivery, including Health Regions, Health Care Maps, and Organizational Contracts for Public Health Action (COAPs). Below is an overview of how the core principles established by this decree can directly influence the care of children with CHD:

## Creation of Health Regions

The decree defines Health Regions as geographical areas in which healthcare actions and services are organized in an integrated manner. However, the effective implementation of regionalization in practice has been a persistent challenge, often resulting in an still unequal distribution of services and resources, with significant gaps in several regions of the country, especially for pediatric cardiovascular surgery**.** This highlights the need for a more creative and contextualized approach to adapt regionalization parameters to Brazilian realities, considering access to treatment as the primary criterion.^13^

## Health Care Networks (RAS)

The decree introduces the concept of Health Care Networks (Redes de Atenção à Saúde – RAS) as a mechanism for coordinating services across different levels of complexity.

## Organizational Contract for Public Health Action (COAP)

The COAP serves as the formal instrument through which federal, state, and municipal governments define their respective responsibilities. In the context of CHD, COAPs can be used to establish region-specific targets for surgical coverage, define and formalize the roles of centers of excellence and regional support hospitals, and determine interstate regulatory mechanisms and proportional financing models.

## Health Care Map

The decree also requires that each federative entity develop a Health Care Map to identify population needs and the availability of existing services.

## National Program for the Comprehensive Treatment of Children with Congenital Heart Disease – 2012

Launched in 2012 by the medical societies: SBC, SBCCV, SBHCI, and SBP, in collaboration with representatives from the MoH, this initiative represented one of the most ambitious attempts to design a comprehensive and specialized public policy for the care of children with CHD in Brazil.

Its central goal was to ensure universal, equitable access to diagnostic, clinical, surgical, and interventional treatment for children with CHD, proposing a robust federative model focused on regional equity and quality of care. It suggested the creation of the Special Secretariat for the Treatment of Children with Congenital Heart Disease (SETRACC) within the MoH, with technical and financial autonomy, and the establishment of a national network of Comprehensive Congenital Heart Disease Treatment Centers (CETICCCs), accredited at different levels of complexity. Additionally, the formation of a national technical advisory board (CTA) comprising medical experts and civil society representatives.

However, despite its technical strength and institutional endorsement, the program was never implemented due to several factors.

### Ordinance No. 20/SCTIE/MS – June 10, 2014

This ordinance made official the inclusion of pulse oximetry, known as the “Little Heart Test” (Teste do Coraçãozinho) as part of universal neonatal screening within Brazil’s Unified Health System (SUS). The decision followed a recommendation from the National Commission for the Incorporation of Technologies (CONITEC), recognizing the importance of this screening tool for the early detection of CHD in newborns.

### Ordinance No. 1,130/GM/MS – PNAISC (2015)

Ordinance No. 1,130, issued on August 5, 2015, established the National Policy for Comprehensive Child Health Care (PNAISC) within the SUS. This policy represented a significant milestone, aligning the principles of comprehensive care, intersectorality, equity, and social participation with the real and dynamic health needs of Brazilian children aged 0 to 9 years.

Although not exclusively focused on CHD, the PNAISC created significant opportunities to strengthen the organization of care for children with heart conditions.

### Ordinance No. 1,727/GM/MS (2017)

Ordinance No. 1,727, issued on July 11, 2017, established the Care Pathway for Children with Congenital Heart Disease within SUS. It was the first federal regulation specifically designed to systematize and integrate the care of children with CHD across the country. Its main objective was to define guidelines and care flows to ensure continuous, timely, and high-quality access to diagnosis, surgical or interventional treatment, and outpatient follow-up for children with CHD, in alignment with the SUS principles of regionalization, equity, and comprehensiveness.

The implementation of Ordinance No. 1,727 was strengthened by the complementary Ordinance No. 3,992/GM/MS (2017), which redefined federal healthcare financing within SUS. This regulation provided crucial financial mechanisms for the Care Pathway, standardizing resource transfer to states and municipalities.

### Ordinance No. 1,940 – June 28, 2018

This ordinance officially incorporated and coded pulse oximetry—the “Little Heart Test” (Teste do Coraçãozinho)—into the SUS Table of Procedures. This measure enabled its routine implementation and reimbursement as part of the national neonatal screening program.

## Situational Diagnostic Project of the Pediatric Cardiovascular Surgery Network in SUS

This project was conducted through a partnership between the Ministry of Health, the Hospital do Coração (HCor), and the Department of Pediatric and Congenital Cardiovascular Surgery (DCCVPed) of the Brazilian Society of Cardiovascular Surgery (SBCCV), funded by the Institutional Development Support Program of SUS (PROADI-SUS).

The initiative aimed for a technical, quantitative, and qualitative mapping of all accredited or active pediatric cardiac surgery centers to support public policies and strategic decisions for network reorganization. Its methodology included on-site technical visits, local team interviews, standardized forms, document audits, and DATASUS data analysis. From 69 centers accredited by late 2018, 2 were deactivated and 13 declined participation (12 without surgical activity), leading to the evaluation of 44 public and philanthropic pediatric cardiac surgery centers across 22 Brazilian states in 2019.

However, critical observations can be made regarding the project’s outcomes. No consolidated national report was officially published, although individual reports were shared with participating centers and a scientific publication by DCCVPed members presented partial findings.^7^ Moreover, limited institutional follow-up and utilization of the data by the MoH, resulting in minimal short-term policy impact.

### Law Project No. 4,700/2019 (amended in 2023)

Introduced by the Brazilian Congress, Law Project No. 4,700/2019 proposes a National Policy for Congenital Heart Disease (CHD) care, with guidelines for early diagnosis, timely treatment, and longitudinal follow-up. A 2023 revision added clearer goals for regionalization, funding, workforce qualification, and equitable access. Currently, the bill remains under legislative review, lacking an approved implementation timeline.

### Law Project No. 4,281/2020

This bill mandates that newborns diagnosed with congenital heart disease through pulse oximetry testing (“Little Heart Test”) be notified to specialized entities. The measure aims to facilitate early referral and appropriate follow-up. The Executive Branch was expected to issue the regulation within 120 days, but this has not yet occurred.

### Decree No. 9,795/2021 – RENASCE Project

On May 17, 2021, Decree No. 9,795 established the RENASCE Project – National Network of Care for Children with Congenital Heart Disease. The project sought to reorganize the national care network with emphasis on regionalization, expansion of installed capacity, workforce training, use of telemedicine, and centralized regulation of surgical and clinical procedures.

The project was conceived with clear and ambitious goals like reducing mortality from CHD by ensuring timely access to surgery and specialized care, reorganizing centers by levels of complexity, establishing a Technical Advisory Chamber, and implement*ing a unified national waiting list.*

Despite some obstacles, the RENASCE Project represented an essential step toward a sustainable, technically sound, and regionally coordinated national policy for congenital heart disease care. Its methodological frameworks and diagnostic insights remain relevant and provide a strong foundation for the resumption of strategic work in 2026.

### Law Project No.15,233/2025 – “*Agora Tem Especialistas*”

The “Agora Tem Especialistas” (Now There Are Specialists) Program, established by Law No. 15,233 and MoH Ordinance GM/MS No. 7,266/2025, is the latest iteration of a policy trajectory. Originating in 2023 as the National Program for Reducing Waiting Lists (PNRF) for elective surgeries, it evolved into the More Access to Specialists Program (PMAE) in 2024, expanding to specialized outpatient and diagnostic services. By 2025, this agenda was consolidated and broadened, now encompassing specialized outpatient care, elective surgeries, radiotherapy, financial credits, SUS digital initiatives, reimbursement, workforce provision/training, and complementary specialized services.

While Cardiology is a priority, enabling expanded consultations, diagnostics, and elective procedures, Pediatric Cardiology and CHD are not specifically mentioned. To strategically embed Pediatric and Congenital Cardiology, the program’s components should align with CHD care pathways, strengthening timely access to specialized outpatient evaluation and echocardiography, improving risk-based regulation and referrals for time-sensitive interventions, and utilizing digital health tools for continuous care and outcome tracking.

Over the last two decades, the comprehensive Care Pathway for Children with CHD has matured, converging initiatives toward a horizontally integrated approach from prenatal diagnosis to long-term follow-up. These efforts prioritize identifying reference centers, training specialized teams, and developing new regional centers to ensure universal, equitable access and standardized care. A critical unmet need remains a robust, centralized national database for reliable statistical and clinical data, including birth defects, CHD surveillance, and mortality/morbidity outcomes to guide policy, an area where DATASUS has historically been insufficient. Equally vital is the institutional recognition of telemedicine for equitable access and regional capacity-building.

The Central Illustration provides more than a chronological list; it maps the implementation lag inherent in the Brazilian CHD care network. By cross-referencing the timeline with the indicators presented in Figure 1, it becomes evident that major regulatory milestones, such as the 2017 National Plan, did not trigger a break in the surgical deficit trend, as shown by Caneo et al.^7^

**Figure 1:** – Number of pediatric cardiac surgeries in. Brazil from 2010 to 2020. Adapted from Caneo et al.7

Number of Pediatric Heart Surgeries in Brazil from 2010 to 2020.

This visual evidence supports the interpretive claim of persistent gaps, as the temporal density of new ordinances contrasts sharply with the stationary clinical outcomes. This decoupling demonstrates that the current policy framework has reached a structural limit, where adding normative layers without operational restructuring fails to improve access.

### Impact of Policies on Care Indicators

The longitudinal analysis reveals a persistent policy-implementation gap. Despite the establishment of the National Policy for High-Complexity Cardiovascular Care in 2004 and the National Plan in 2017, the national surgical deficit has remained critically high.^14,16^ According to Barreto et al.,^17^ the deficit in corrective surgeries was 64.27% in 2010 and only marginally improved to 51.46% by 2019, before rising again during the COVID-19 pandemic, what is graphically represented on Figure 2. Regional disparities remain a structural bottleneck: the North region consistently presents the highest cumulative deficit (75.87%), contrasting with the South (47.32%).^12,17^ Furthermore, while the mandatory “Little Heart Test” in 2014 improved early diagnosis, data indicate it was not followed by a proportional expansion of fully-operational surgical centers, as demonstrated in the Situational Diagnosis from 2019.^7^

**Figure 2:** – Trends in Surgical Volume for Congenital Heart Disease in Brazil. Adapted from Barreto et al.17 The graph illustrates the progressive increase in the absolute number of CHD surgeries, reaching a peak of procedures in 2019 (pre-pandemic).

Regarding mortality trends, in 2024, Cohen et al.^18^ identified a significant shift starting in 2018, three years after the mandatory implementation of neonatal pulse oximetry screening (Little Heart Test). However, this decline was not uniform; while national rates showed a downward trend, the North region remained an outlier with an increasing mortality rate during the same period.^18^

The findings demonstrate a significant decoupling between normative progress and clinical outcomes in Brazil. The content of policies, such as Ordinance No. 1,727/2017, was technically robust, but the implementation process faced critical bottlenecks. As highlighted by Soares et al.^16^ the goal of increasing care by 30% per year remains largely unfulfilled due to chronic underfunding and the lack of a specialized workforce.^16^ The transition to global-sum financing (Ordinance No. 3,992/2017) often diluted resources intended for CHD, explaining why regionalization remains a theoretical goal rather than a practical reality. The lack of a unified clinical database, beyond administrative billing data (DATASUS) remains a major barrier to identifying specific nodes of failure within the Care Pathway, preventing outcome-driven adjustments to the national strategy.

The correlation between legislative milestones and care delivery indicators reveals a significant execution gap in the Brazilian CHD care network. As illustrated in Figure 2, although there was a progressive increase in the absolute volume of surgeries, peaking at 9,956 procedures in 2019, this expansion was insufficient to offset the structural deficit. Data synthesized from Barreto et al.,^17^ and Miana et al.^12^ show that the national surgical deficit remained chronically above 50% for the last decade, as observed in Figure 3, indicating that the system reached a structural plateau despite the normative advances of the 2017 National Plan.

**Figure 3:** – Longitudinal Analysis of National Deficit and Mortality Rates from Congenital Heart Disease in Brazil. Adapted from Barreto et al.17 The national surgical deficit remained chronically above 50%, demonstrating a structural plateau in care delivery capacity.

### Proposal for a “New” Model

The assertion that Brazil requires a robust, centralized national database for CHD is evidenced by the inherent limitations of the current administrative systems. As demonstrated by Miana et al.,^12^ the SIH/SUS lacks clinical granularity, failing to distinguish between anatomical complexities or to track long-term functional outcomes. Furthermore, Barreto et al.^17^ highlighted that reliance on secondary billing data can lead to underreporting and coding inconsistencies, masking the true surgical deficit. The absence of a clinical registry, similar to the STS-CHSD in North America, prevents the implementation of outcome-based financing and risk-adjusted quality monitoring, which are standard in high-performance CHD networks.

The proposed model is an evidence-based framework constructed through a triangulation of three analytical pillars: (1) identification of implementation failures in Brazil; (2) international benchmarking of high-performance networks like the STS-CHSD; and (3) stakeholder consensus between the MoH, SBC, SBCCV, SBHCI, and SBP with supervision of CHL. It shifts from a normative-heavy approach to an operational-driven strategy.

To achieve this operational shift, the model is structured upon the following strategic pillars:

1. Regionalization of CHD care through the definition of integrated macroregional networks linked to centers of excellence.
2. Implementation of retraining and teletraining programs for regional hospital teams, strengthening the integration via shared protocols and teleconsultation.
3. Restructuring of the National Center for High-Complexity Regulation (CNRAC) for CHD, ensuring greater efficiency and transparency in referral and transport logistics.
4. Establishment of a regulatory model combining federal planning with decentralized regional implementation.
5. Real-time monitoring of outcomes and quality indicators, including the creation of an independent database, separate from DATASUS, for evaluating CHD-related health metrics.
6. Optimization of resource utilization across the CHD care continuum within the SUS.

We recommend that the MoH lead a national consultative planning process with stakeholders to define standards, set priorities, and develop a roadmap for Brazil’s pediatric cardiac health system. This aims to forge a national strategy and plan for workforce, infrastructure, capacity, and financing in pediatric and congenital heart services. Following this, steps to improve screening, referral, and surgical/medical treatment of pediatric and congenital heart disease are outlined, aligning with national strategic priorities and contributing to the Sustainable Development Goals to reduce childhood mortality from these conditions.

### 1. Regionalization of Care

The first step involves mapping healthcare capacities by macroregion, defining referral and counter-referral flows between local units and centers of excellence, and establishing regional and national service delivery targets

### 2. Partnerships with Centers of Excellence

National centers of excellence will be selected based on technical criteria—such as case volume, outcomes, multidisciplinary teams, academic involvement, and strategic location—to establish partnerships for retraining, technical supervision, and regulatory support. Currently, a distinction exists between Assistance Units and Reference Centers. The proposal is to redefine these centers based on performance and quality criteria, maintaining or revising nomenclature as needed. A national coordinating technical nucleus will also be created, ensuring representation from both assistance and reference centers.

### 3. Retraining and Tele training

Building upon existing Ministry of Health initiatives, including those implemented at Instituto do Coração (InCor–HCFMUSP) and HCor, a continuous (in-person and remote) capacity-building program will be developed, focusing on all stages of CHD care. Support for primary care professionals in recognizing and managing congenital heart disease.

### 4. Restructuring of Regulation and Patient Transfer

Although mechanisms such as CNRAC and CRACs already exist, the proposal includes a redesign of the triage and authorization process. Regulation will be fully digitized, based on objective clinical criteria and maximum response times. A prioritization system will be created that accounts for clinical risk and geographic accessibility. Critical patients will have access to air medical transport via public–private partnerships (PPP).

### 5. Federal Governance with Regional Execution

Policy, funding, and performance evaluation will remain centralized under the MoH, while operational implementation will occur through articulated regional networks. A National Technical Advisory Chamber will be established, with consultative and deliberative functions.

To ensure cross-level coordination, a National CHD Steering Committee (NCSC) shall be established:

- Strategic Leadership: The MoH holds primary accountability for funding, national regulation via CNRAC, and policy oversight.
- Technical Governance: A permanent Technical Advisory Chamber (representatives from SBC, SBCCV, SBHCI, and SBP) will define clinical protocols and accreditation standards.
- Independent Auditing: CHL will act as an international technical partner for the independent certification of ‘Centers of Excellence’ based on global performance benchmarks.

### 6. Data Access and Monitoring

Improving data accessibility and integration is essential to track patient care trajectories within SUS. Starting in 2026, all patients diagnosed with congenital heart disease will have their diagnosis linked to their national identification number (CPF), enabling longitudinal tracking of hospitalizations, procedures, clinical treatments, and outcomes. This approach will enable real-time surveillance, data-driven planning, and the development of a robust national epidemiological database. The assertion that Brazil requires a robust, centralized national database is grounded in the clinical insufficiency of current systems.

### 7. Counter-Referral and Regulation Specificities

Effective organization of CHD care requires not only defining reference centers and centralizing regulation, but also formalizing counter-referral processes and ethical safeguards to ensure equitable, transparent access.

In cases where a patient is deemed ineligible for transfer or surgery, a mandatory teleconsultation will be held with the family, during which the clinical reasoning for non-indication will be explained and formally recorded. Once stabilized, patients shall be referred back to their originating units through a formalized counter-referral pathway to ensure continuity of care and prevent overloading tertiary centers.

### 8. International Partnerships and Cooperation

There is an opportunity for technical collaboration with the international organization Children’s HeartLink (CHL), which can assist in selecting, training, and certifying both centers of excellence and regional training hubs in accordance with global standards for quality, sustainability, and impact.

### 9. Expected Outcomes

- Reduction in waiting lists for CHD surgeries.
- Decrease in infant mortality from treatable cardiac causes.
- Expansion of accredited and qualified regional centers.
- Increased regional capacity and reduced need for patient transfers.
- Enhanced clinical and regulatory decision-making.
- Improved outcome indicators and long-term follow-up data.

### 10. Proposed Stakeholders and Partners

- Ministry of Health – DAET / SAES
- National Council of Health Secretaries (CONASS)
- National Council of Municipal Health Secretariats (CONASEMS)
- Accredited congenital cardiac surgery programs
- Brazilian Society of Cardiology (SBC), through the Department of Pediatric Cardiology (DCC/SBC)
- Brazilian Society of Cardiovascular Surgery (SBCCV), through its Department of Pediatric and Congenital Cardiovascular Surgery (DCCVPed)
- Brazilian Society of Hemodynamics and Interventional Cardiology (SBHCI)
- Brazilian Society of Pediatrics (SBP)
- Children’s HeartLink (CHL)

### 11. Implementation Timeline

**Phase 1 – Planning and Structuring (until December 2027):**

- Define National Centers of Excellence based on objective technical criteria.
- Select and accredit Regional Training and Education Centers.
- Establish agreements with state and municipal health departments regarding referral flows and reference centers.
- Train regional services to register patients in the national CNRAC-regulated waiting list.

**Targets by the end of 2027:**

- All reference centers defined and accredited.
- All services qualified to register patients in the national platform.

**Phase 2 – Progressive Implementation and Centralized Regulation (2029):**

- Launch of the National Unified Regulation System under CNRAC.
- Establishment of a national prioritization metric based on waiting time and case complexity.
- Automatic reassignment of urgent cases not admitted within 48 hours.
- Definition of each center’s capacity according to complexity profile.
- Mandatory teleconsultation for non-transferable or non-surgical cases.
- Mandatory counter-referral following clinical stabilization.

**Targets by the end of 2029:**

- Nationwide implementation of the program.
- 80% of CNRAC requests resolved within 48 hours.
- Fully operational, transparent, and technically driven regulatory system.
- Functional counter-referral flows across all regions.
